# Composite Biomimetic Multi-Subsoiler for Drag Reduction and Wear Resistance Simulation and Experimental Validation

**DOI:** 10.3390/biomimetics10120793

**Published:** 2025-11-21

**Authors:** Xiaoyang Wang, Jinguang Li, Junyan Liu, Le Yang, Fancheng Dai, Chanjuan Long, Lijun Zhao

**Affiliations:** 1 School of Mechatronics and Information Engineering, Chongqing College of Humanities, Science and Technology, Chongqing 401524, China; t1232185@cqrk.edu.cn (X.W.);; 2 Intelligent Manufacturing Technology Innovation Center in Hechuan District, Chongqing 401524, China; 3 State Key Laboratory of Intelligent Agricultural Power Equipment, Luoyang 471000, China; 4 School of Mechatronics Engineering, Henan University of Science and Technology, Luoyang 471003, China; 5 School of Intelligent and Manufacturing Engineering, Chongqing University of Arts and Sciences, Chongqing 402160, China

**Keywords:** subsoiler, bionic optimization, bionics design, response surface analysis (RSA), discrete element method, orthogonal experiment

## Abstract

In the process of operating subsoiling implements on sloping red soil in Southwest China, the subsoiler tip faces significant challenges due to strong soil adhesion and severe compaction. By employing engineering bionics, integrating bionic geometric structures and surfaces, this study focuses on the subsoiler tip and designs four types of bionic geometric surface structures: bionic convex hull, bionic micro-spike convex hull, bionic scales, and bionic micro-spike scales. Finite element force analysis and discrete element simulation experiments reveal that bionic surfaces and geometric structures exhibit significant advantages in terms of total deformation, equivalent elastic strain, and stress. These structures are less prone to deformation and fracture under loads, demonstrating a stronger bearing capacity. A discrete element simulation analysis indicates interference phenomena among the subsoilers during multi-subsoiler operations. Based on bionic multi-subsoiler implements, optimized designs were developed through discrete element simulations and soil bin tests. The optimized bionic multi-subsoiler implement features a micro-spike convex hull surface, with micro-spike scale surfaces arranged equidistantly along the edge corners of the shovel face: six on each side wing and three in the middle. The optimal operating parameters were a subsoiling speed of 1.25 m/s, an entry angle of 23.917°, and an entry depth of 280.167 mm. The relative errors between the simulated and experimental values for the soil looseness and soil disturbance coefficients were 19.7% and 18.1%, respectively. The soil bin test results showed soil looseness and soil disturbance coefficients of 19.5% and 17.6%, respectively. At this point, the resistance reduction and wear resistance performance were optimal. This study proposes a bionic design approach for reducing resistance and enhancing wear resistance during the subsoiling process in the viscous red soil of Southwest China, providing a reference for the design and development of new equipment for working in this soil environment. This study is the first to implement a composite biomimetic surface—combining crayfish-like micro-spike convex hulls and sandfish-like micro-scale scales—on multi-shank subsoiler tips, and to validate it using FEA, DEM, and soil tank testing. Under an optimized configuration and operating conditions, the mean particle disturbance velocity increased from 1.52 m/s to 2.399 m/s (+57.8%), and the simulation/experiment relative errors for the soil loosening and disturbance coefficients were approximately 1.03% and 2.84%, respectively. These results demonstrate an engineering-acceptable trade-off between disturbance efficiency and wear resistance and indicate a clear potential for industrial application.

## 1. Introduction

To address the challenge of high subsoiling resistance in northern China subsoiling operations, Zhao Shuhong et al. [1] optimized the design of the subsoiler blade curve, creating a subsoiler that reduces resistance. Yuan Jin et al. [2] proposed an active lubrication and soil improvement composite operation scheme for a curved subsoiler, targeting low-moisture-content and high-compaction soils to tackle issues of high resistance, high traction energy consumption, and low operational efficiency. In the context of the protective tillage of southern red soil, research on subsoiling machinery is limited and still at the shallow tillage stage. The long-term traditional tillage methods in southern China have led to the upward movement of the plow pan and increasingly severe soil impoverishment, resulting in the poor efficiency of traditional subsoiling operations [3]. The research on mechanized subsoiling technology mainly depends on high-efficiency and high-strength implements compatible with high-power tractors. In the study of high-strength implements, the subsoiling process on sloping red soil in Southwest China primarily exerts forces on the subsoiler’s handle and tip. Due to the challenges of insufficient supporting power, high tillage resistance, and severe wear during subsoiling, traditional subsoiling implement designs can no longer meet the requirements for resistance reduction. Designing bionic geometric surface structures with an outstanding resistance reduction has become a development trend for efficient resistance reduction and wear resistance in mechanical soil-contact components [4]. Bionics research has a significant impact on solving the practical engineering problem of high resistance. Garibaldi-Márquez et al. [5] extracted the arc of the mole rat’s claws and applied it to the subsoiler blade, improving the resistance reduction capability of the subsoiling implement by refining the blade arc. Zhang Zhihong et al. [6] addressed the issues of traditional subsoiling implements’ difficulty in breaking soil and high tillage resistance by using the head of the *Scincus scincus* as a bionic prototype. They employed reverse engineering technology to extract its unique geometric features and applied the quantified geometric structure characteristics to the design of the subsoiler tip, aiming to reduce subsoiling resistance and energy consumption. Zhou, D et al. [7] proposed a design method for forced vibration subsoiling to reduce resistance and consumption. The vibration digging parameters of Antlion larvae were obtained using a high-speed camera, and a forced-vibration subsoiler handle and six different shovel tips were designed through the non-smooth resistance-reducing surface design method. The resistance-reducing and consumption-reducing effects were verified in the field subsoiling experiment. Considering the increasing trend of mechanized farming areas in southern China regions, traditional tillage has led to severe soil compaction, the high adhesion of red soil, and significant soil adhesion during subsoiling, resulting in a high tillage resistance [8]. Investigating the challenges of high resistance and severe wear in traditional subsoiling operations on red soil is of great significance. In summary, the anti-drag and anti-friction performances of low-resistance animals in soil have always been the focus of study for soil-plowing devices [9]. Currently, there is extensive research on bionic designs for single-subsoiler implements, but integrated bionic design studies for multi-subsoiler implements are limited. By testing traction resistance and observing sudden disturbances, subsoiling power consumption can be analyzed. To address the challenge of high resistance during subsoiling on sloping red soil, this study utilizes the excellent resistance reduction and wear resistance characteristics of burrowing organisms and soil. Using engineering bionics [10], an optimized design for multi-subsoiler implements with a superior resistance reduction and wear resistance was developed, solving the problems of high resistance and severe wear during subsoiling operations in Southwest China.

In order to position this work within the current international development of subsoiler equipment and to better justify the necessity and opportunity for the present study, we have expanded the introduction to cite recent advances in subsoiler design and testing. Recent works illustrate both the active global research effort in subsoiler optimization and the practical demands for improved implements. For example, a similarity-based DEM simulation combined with soil bin testing has been used recently to optimize subsoiler geometry for cohesive–frictional soils, demonstrating a rigorous pathway from numerical modeling to laboratory validation [11]. Practical guides and field-oriented design reports further document user needs and operating constraints for successful subsoiling in diverse agronomic contexts [12]. In addition, mathematical modeling and structural optimization studies have quantified the relations between subsoiler geometry and specific resistance, providing analytical support for targeted design modifications [13]. Taken together, these international studies confirm a clear opportunity and an urgent need for integrated multi-shank solutions that combine novel surface geometries with deployable implement arrangements. Accordingly, the objective of the present study is to develop and validate a composite biomimetic multi-subsoiler—combining crayfish- and skink-inspired microstructures—and to demonstrate through combined FEA, DEM, and soil bin testing that such a design can substantially reduce draft and improve wear performance under sloping red soil conditions.

To address the high draft and severe wear encountered by conventional multi-shank implements in sloping red soil, this study proposes and validates an integrated biomimetic multi-shank design that combines crayfish (*Procambarus clarkii*) micro-spike convex hulls with skink (*Scincus scincus*) micro-scale surfaces and optimizes their arrangement across multiple shanks. Using combined FEA, EDEM, and soil bin validation, the optimized multi-shank arrangement demonstrates a quantitatively superior performance: the mean particle disturbance velocity increased from 1.52 m/s (baseline) to 2.399 m/s (optimized), a ≈ 57.8% increase; the simulation vs. soil bin relative errors for soil looseness and disturbance are ≈1.03% and ≈2.84%, respectively. Unlike prior work focused on single-tip biomimetics, this study’s novelty lies in the composite use of two distinct biological microstructures within a multi-shank configuration and the explicit evaluation of inter-shank interference and wear distribution—providing direct, quantifiable evidence for engineering implementation.

## 2. Design of the Bionic Geometric Structure Surface of the Subsoiler Tip

**Figure 1:**
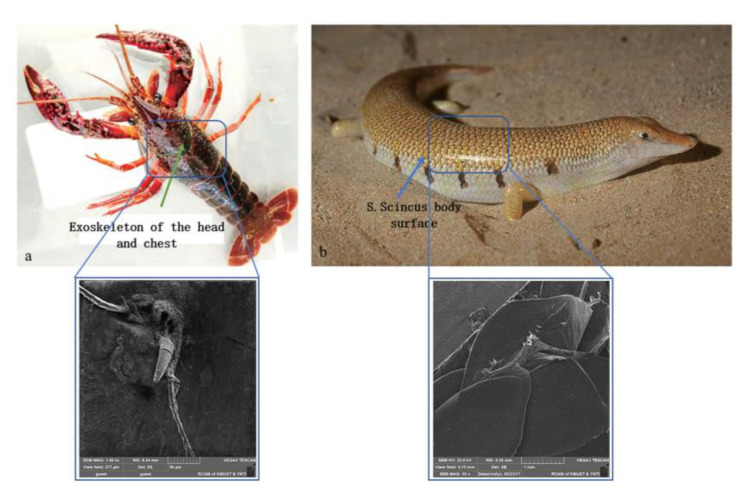
Microscopic structure of biological surfaces under electron microscopy: (**a**) cephalothoracic exoskeleton of *Procambarus clarkii*; (**b**) body surface of *Scincus scincus*.

**Figure 2:**
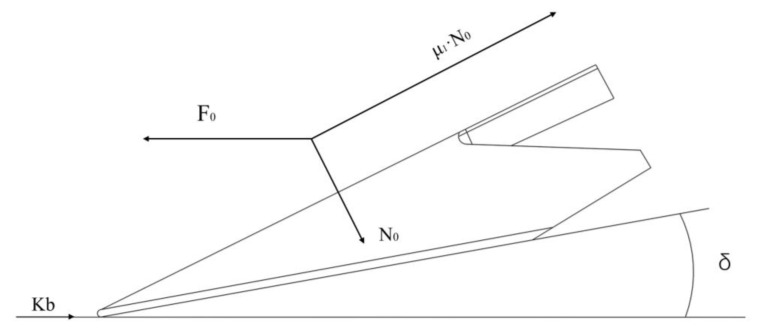
Force analysis of Subsoiler.

Based on the living environments of *Procambarus clarkii* and the *Scincus scincus*, which are, respectively, humid and gravelly, both exhibit excellent resistance reduction capabilities. The living environments and resistance reduction characteristics of *Procambarus clarkii* [14] and the *Scincus scincus* [15] during movement provide a theoretical foundation for selecting bionic prototypes. According to the experimental studies by Raabe [16,17,18], Sachs [19], and Romano [20] on *Procambarus clarkii*, and the experimental results by Friederike Saxe [21] on the *Scincus scincus*’s epidermis, as observed through scanning electron microscopy (SEM) (Figure 1), it was found that the primary resistance reduction structures of *Procambarus clarkii* are the micro-spike convex hull geometric structures on the cephalothorax exoskeleton, while the primary wear-resistant surfaces of the *Scincus scincus* are the micro-spike scale geometric surfaces on the dorsal body surface. As shown in Figure 2, the SEM microstructures were obtained. By integrating the bionic structures and surfaces of both organisms, a bionic geometric surface with an excellent resistance reduction performance was designed. After optimization, the designed bionic geometric surface was applied to the subsoiler tip, and its resistance reduction performance was experimentally evaluated.

## 3. Modeling and Finite Element Analysis of the Bionic Subsoiler

The force analysis of the subsoiler head and shank was conducted. The subsoiler is divided into two parts: the shovel head and the shovel shank. Therefore, the total working resistance of the subsoiler can be considered as the sum of the forces on these two parts, i.e., $F=F_{0}+T$ represents the force acting on the shovel head, and T represents the force acting on the shovel shank. As shown in Figure 2, the forces on these two parts were calculated separately:

(1) $$F_{0}=N_{0}sin\delta +\mu_{1}N_{0}cos\delta +kb$$

**Figure 3:**
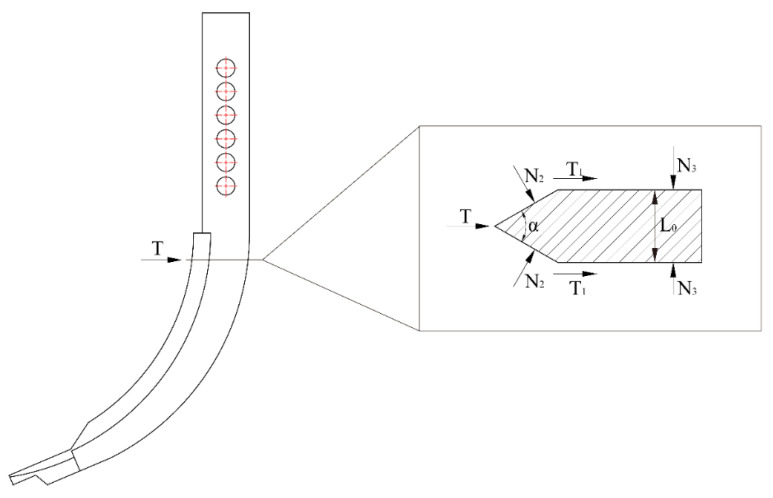
Force analysis of Subsoiler shovel shaft.

Let the horizontal traction force be $F_{0}$ and the normal pressure in the normal direction be $N_{0}$. These forces are generated by the soil’s reaction force on the shovel head, as the shovel head exerts a thrust on the soil, making the soil the force-bearing object. Let the cutting resistance of the soil per unit width be k and the width of the shovel blade be b. Let the entry angle of the shovel head be δ, with δ valued at 23.4°. Let the friction coefficient between the shovel surface and the soil be $\mu_{1}$, with a reference value of 0.6 according to Zhang Zhao et al. [22]. The analysis shows that a medium-sized subsoiler shank is used in this study. The forces on the shovel shank can be decomposed into several components, resulting in the force analysis diagram of the shovel shank during subsoiling. The equipment was designed with reference to Chinese Machinery Industry Standards and was procured from Chongqing Huashidan Agricultural Equipment Manufacturing Co., Ltd., Chongqing, China. as shown in Figure 3.

(2) $$\begin{array}{l} P_{1}=N_{2}sin\frac{\alpha }{2} \end{array}$$

(3) $$\begin{array}{l} T=N_{2}cos\frac{\alpha }{2} \end{array}$$

(4) $$\begin{array}{l} T_{1}=2N_{3}\mu_{1} \end{array}$$

(5) $$\begin{array}{l} F_{2}=2\left(P_{1}+T_{1}+T\right) \end{array}$$

(6) $$\begin{array}{l} F_{2}=2N_{2}sin\frac{\alpha }{2}+2N_{2}\mu_{1}cos\frac{\alpha }{2}+2N_{3}\mu_{1} \end{array}$$

**Figure 4:**
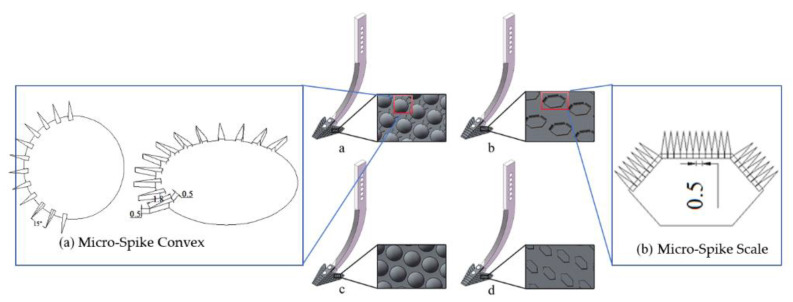
Bionic subsoiler model. (**a**) Micro-spike convex, (**b**) micro-spike scale, (**c**) spike convex, (**d**) spike scale.

According to equation, modifying the subsoiler shank model by controlling the opening angle of the shovel shank blade and the entry angle can effectively control the resistance encountered during subsoiling operations. Four bionic geometric structures and surfaces were designed: a bionic convex hull structure, bionic micro-spike convex hull structure, bionic scale surface, and bionic micro-spike scale surface. These designs were applied to the surface of the subsoiler. The selected dimensions for the convex hull are a base diameter of 12 mm and a height of 1.8 mm, with micro-spikes having a height of 1.8 mm and a thickness of 0.5 mm. The bionic geometric structures were set to cover 45–55% of the surface area of the subsoiler tip, maintaining a uniform area ratio, as shown in Figure 4.

## 4. Discrete Element Simulation of the Operation Process of the Bionic Multi-Subsoiler Implement

### 4.1. Establishment of a Subsoiling Implement Model

**Figure 5:**
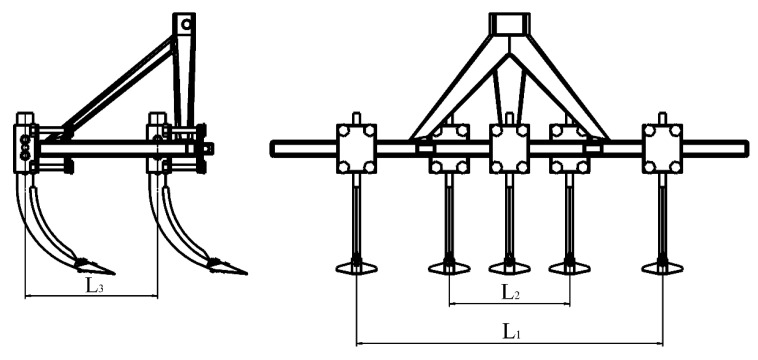
Multiple subsoiler implement models.

According to the operational performance, reliability indicators, and assembly requirements specified in the national standard GB/T 24676-2021 [23]. A simplified frame model that meets the assembly requirements of the subsoiler was drawn, and the subsoiling device was assembled. The spacing between the front row of subsoilers, $L_{1}$, is 1154 mm, the spacing between the rear row of subsoilers, $L_{2}$, is 454 mm, and the spacing between the front and rear rows of subsoilers, $L_{3}$, is 500 mm, as shown in Figure 5.

In the discrete element simulation tests, the required model parameters mainly include the intrinsic material properties, such as Poisson’s ratio, shear modulus, and material density. This study focuses on analyzing the force variations and particle velocity distribution around subsoiler tips with different bionic surface structures under identical operating conditions. Therefore, only the geometric structure of the subsoiler tip surface was varied during the simulations. The simulation parameters were determined with reference to the works of Deng Jiayu et al. [24] and Xuezhen Wang et al., who established soil parameters for subsoiler simulations, and were further adjusted based on the physical property measurements of the test soil obtained by our research group. The resulting discrete element parameter scheme is shown in Table 1. Considering the cohesive characteristics of clay soil, the JKR (Johnson–Kendall–Roberts) contact model was adopted, which accounts for the adhesive forces between particles through surface energy. According to Wu Tao et al. [25,26], the surface energy was set to 7.91 J/m^3^ at a soil moisture content of 16.2%. The shovel body material was 65 Mn, conforming to the national standard, and its shear modulus was calculated using Equation (7).

(7) $$\begin{array}{l} G=\frac{E}{2\left(1+\mu \right)} \end{array}$$

### 4.2. Establishment of a Soil Model

The discrete element simulation experiment was conducted in a soil bin model with dimensions of 2000 mm × 8000 mm × 500 mm. In the pre-processor, settings were configured for the particle model, particle drop speed, subsoiler movement direction and speed, and particle factory [21,22,24]. The analysis focused on the interaction between five different shovel tips and the soil, examining the effects of different shovel tip surfaces on the forces exerted on soil particles, the changes in particle movement speed after being subjected to force, and the variations in traction force during the movement of the subsoiler, as shown in Table 1.

### 4.3. Establishment of the Discrete Element Models for the Interaction Between Single-Shovel and Multi-Subsoiler Implements and Soil

**Figure 6:**
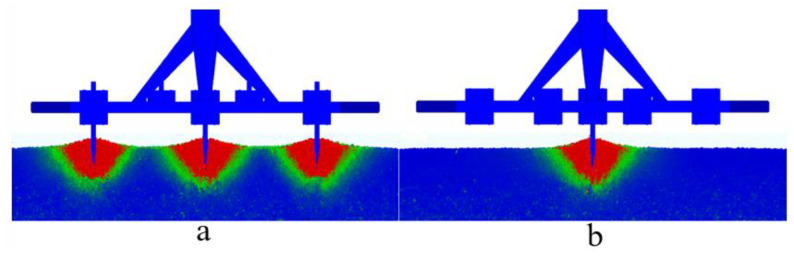
Interference phenomenon of interaction between subsoiling implements and soil. (**a**) Multi-shank configuration; (**b**) Single-shank configuration.

The models of subsoiling devices with multiple subsoilers and a single subsoiler were imported into EDEM. The centroid of the subsoiler frame model was defined at (0, 2500 mm, 350 mm). Using the centroid of the subsoiling device as the origin, a forward speed of 1 m/s was added. The analysis under the same motion parameters and soil model revealed that, during the operation of the multi-subsoiler implement with the soil, interference phenomena occurred between the subsoilers. The soil particle speed at the central position was the highest, and the subsoiler exhibited a significant contact energy. The interaction between the subsoiler and the soil on the left and right sides showed asymmetry. The study focused on analyzing the force changes and wear during the operation of the subsoiling implement with the soil, using the multi-subsoiler implement as the research model, as shown in Figure 6.

### 4.4. Discrete Element Simulation Experiment and Analysis

**Figure 7:**
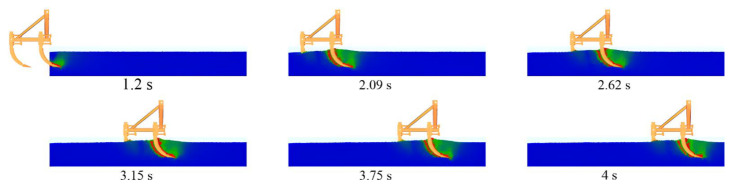
Force diagrams of soil particles at different time intervals.

Using the multi-subsoiler implement as the research object, during the simulation, soil particles were generated by the factory from 0 to 1 s. After the particles completed their fall, the subsoiler entered the soil model at a speed of 1 m/s from 1 s to 4 s, and then moved horizontally at a speed of 1 m/s for 3 s. This experiment primarily observed the interaction effects between the shovel tip surface and the soil force, without considering factors such as entry angle and entry method. By changing the surface structure of the shovel tip and analyzing the speed and force changes in soil particles at different times, the cumulative change in tangential energy of the subsoiler was used to analyze the performance of each group of shovel tips, as shown in Figure 7.

Please refer to the data chart, the above experimental results were post-processed. The entire experiment was divided into 20 steps, exporting a total of 20 segments of CSV (Comma Separated Values) data, which were then visualized using conventional line charts for intuitive comparison. Under traditional subsoiler operations, the average soil particle speed on the shovel tip surface was 1.52 m/s, with a maximum of 2.32 m/s and a minimum of 1.19 m/s. Under the action of the bionic convex hull subsoiler, the average soil particle speed was 1.88 m/s, with a maximum of 2.46 m/s and a minimum of 1.29 m/s. Under the action of the bionic micro-spike convex hull subsoiler, the mean soil particle speed was 1.92 ± 0.30 m/s (mean ± SD, *n* = 20), with a maximum of 3.19 m/s and a minimum of 1.45 m/s. Under the action of the bionic scale subsoiler, the average soil particle speed was 1.67 m/s, with a maximum of 2.18 m/s and a minimum of 1.30 m/s. Under the action of the bionic micro-spike scale subsoiler, the average soil particle speed was 1.64 m/s, with a maximum of 2.33 m/s and a minimum of 1.14 m/s.

Please refer to the data chart operations, the average force on soil particles on the shovel tip surface was 1.54 N, with a maximum of 2.76 N and a minimum of 0.94 N. Under the action of the bionic convex hull subsoiler, the average force on soil particles was 3.90 N, with a maximum of 7.72 N and a minimum of 1.29 N. Under the action of the bionic micro-spike convex hull subsoiler, the average force on soil particles was 3.35 N, with a maximum of 5.29 N and a minimum of 1.89 N. Under the action of the bionic scale subsoiler, the average force on soil particles was 3.00 N, with a maximum of 5.33 N and a minimum of 1.45 N. Under the action of the bionic micro-spike scale subsoiler, the average force on soil particles was 2.39 N, with a maximum of 6.59 N and a minimum of 1.15 N.

**Figure 8:**
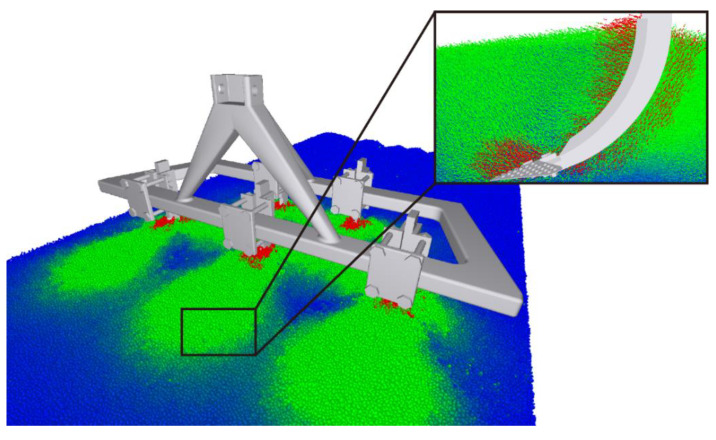
Velocity of soil particles during subsoiling process.

**Figure 9:**
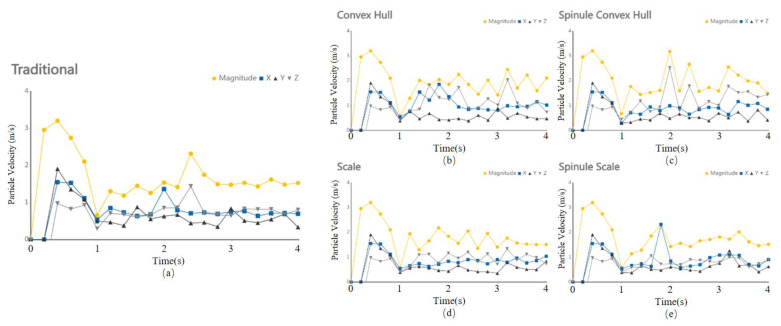
Velocity of particles during the collision between five types of subsoiling implements and soil. (**a**) Particle velocity on the surface of a conventional subsoiler tip; (**b**) Particle velocity under the action of a bionic convex hull structure subsoiler; (**c**) Particle velocity under the action of a bionic micro-spine convex hull structure subsoiler; (**d**) Particle velocity under the action of a bionic scale structure subsoiler; (**e**) Particle velocity under the action of a bionic micro-spine scale structure subsoiler.

As shown in Figure 8 and Figure 9, please refer to the data chart, it was found that the subsoiler with the bionic micro-spike convex hull structure had the best soil disturbance effect, with an average value of 1.92 m/s. The subsoiler with the bionic scale structure showed relatively stable soil disturbance effects but poorer performance, with an average value of only 1.67 m/s. In terms of the force on soil particles during subsoiling operations, the subsoilers with bionic micro-spike convex hull and bionic scale structures exhibited relatively stable forces on soil particles, with average values of 3.90 N and 3.35 N, respectively. The subsoiler with the bionic convex hull structure exerted a larger force on the soil, with an average value of 3.90 N. A moderate force on the soil during subsoiling operations can better complete the subsoiling task, but excessive force may compact the soil, affecting the subsoiling effect and hindering plant growth. When analyzing performance using particle speed as an indicator, the soil force should not be too small or too large.

### 4.5. Performance and Wear Analysis of Various Subsoilers During Subsoiling Operations

**Figure 10:**
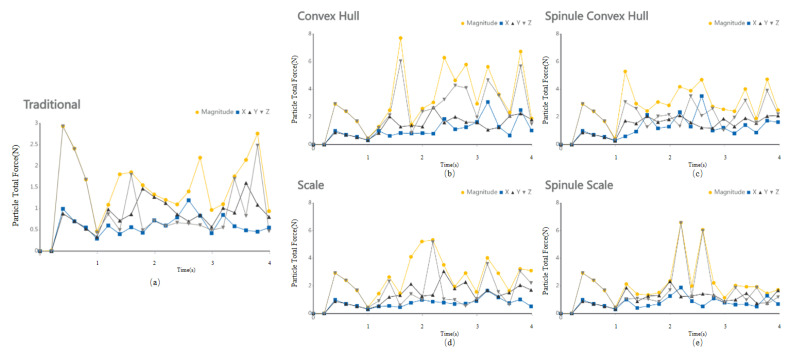
Forces on particles during the collision between five types of subsoiling implements and soil. (**a**) Particle force on the surface of a conventional subsoiler tip; (**b**) Particle force under the action of a bionic convex hull structure subsoiler; (**c**) Particle force under the action of a bionic micro-spine convex hull structure subsoiler; (**d**) Particle force under the action of a bionic scale structure subsoiler; (**e**) Particle force under the action of a bionic micro-spine scale structure subsoiler.

The discrete element particle velocity data and shovel wear data were integrated, with cumulative contact force referenced from Li Jicheng et al. [27]. The standard error was used as a statistical indicator to measure the uncertainty difference between the sample mean and the population mean. When comparing the five sets of data, as shown in Figure 10, the particle velocity, force conditions, and shovel force conditions during subsoiling operations can be observed, especially considering the bionic structure of micro-spike convex hulls. The results indicate that, under the same operating conditions, subsoiling tools with the bionic structure of micro-spike convex hulls exhibited a higher particle velocity, greater force conditions, and a reduced force on the subsoiler. By loading the Relative Wear model’s discrete element data, the wear conditions of a traditional subsoiler and subsoilers with different bionic structures were compared. The results showed that the tip wear of a traditional subsoiler was mainly distributed on the shovel surface and edge corners, while the wear of subsoilers with bionic scale structures was mainly on the edge corners with rounded parts. Additionally, the wear of subsoilers with bionic convex hull structures was concentrated on the convex hull surface facing the soil and the edge corners of the shovel surface. Subsoilers with bionic micro-spike scale structures exhibited the least tangential cumulative contact with the soil during operations and demonstrated relatively stable performance.

### 4.6. Optimization of the Subsoiler Structure

**Figure 11:**
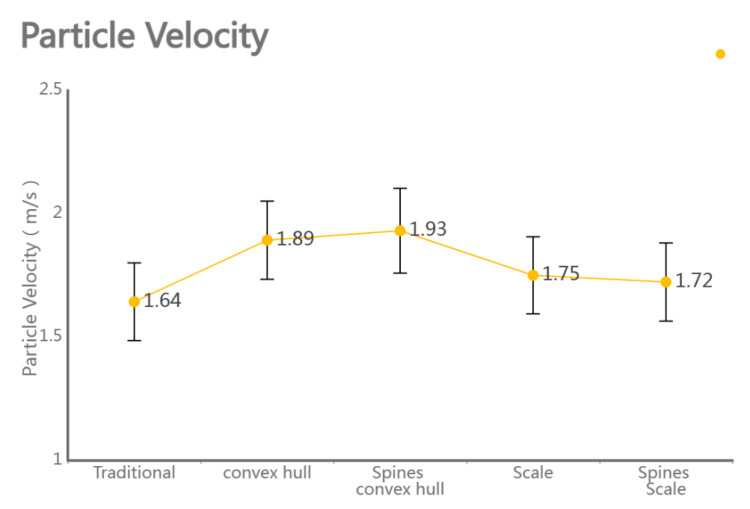
Comparison of particle velocities in five groups.

**Figure 12:**
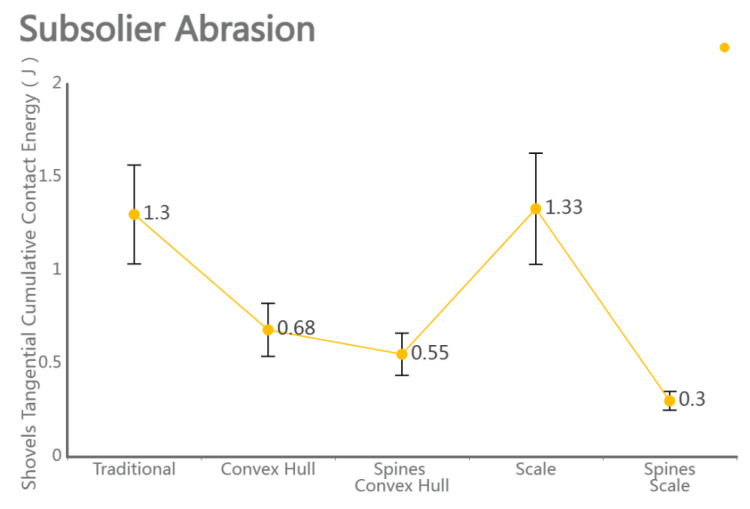
Comparison of wear amounts for five groups of subsoiling implements.

**Figure 13:**
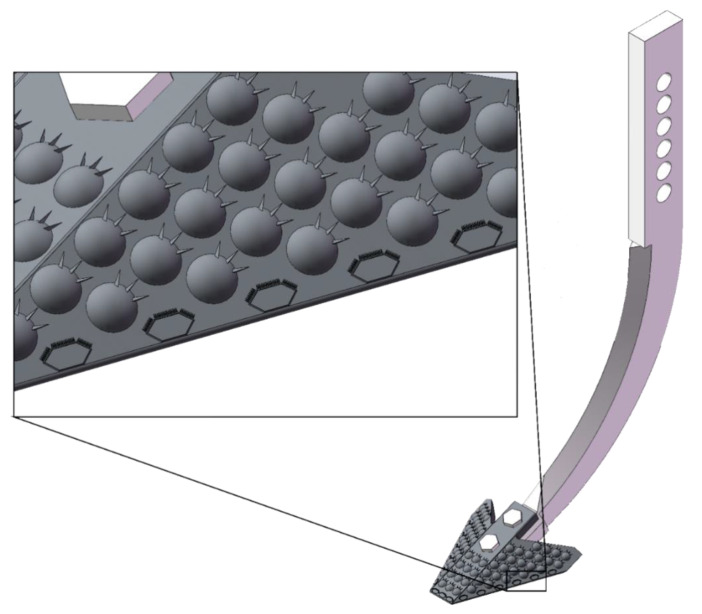
Optimized design of subsoiler model.

**Figure 14:**
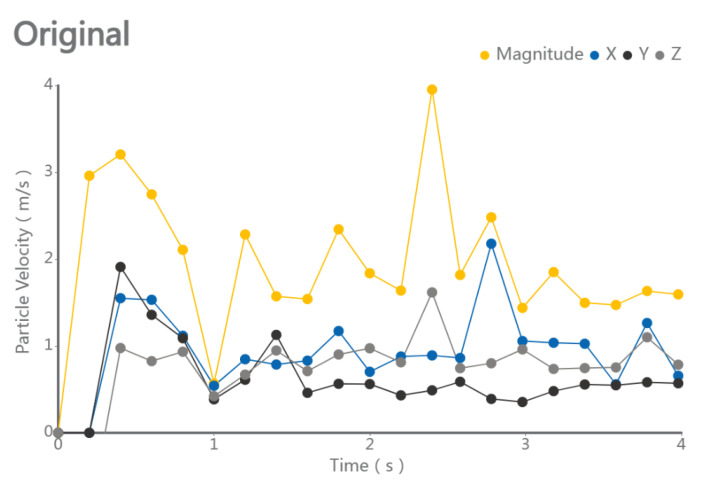
Changes in particle velocity after optimization.

**Figure 15:**
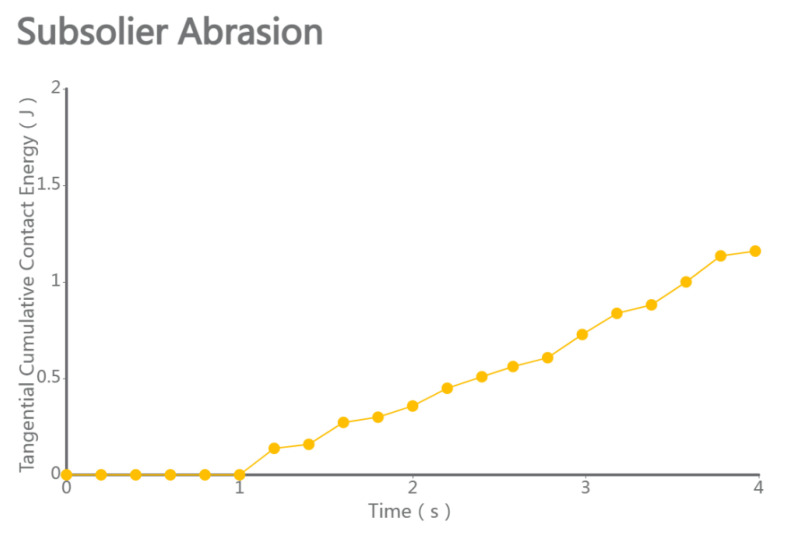
Tangential energy of subsoiler blade.

**Figure 16:**
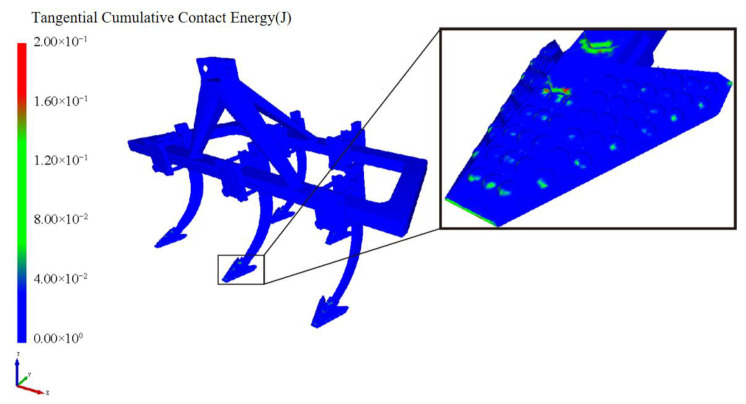
Cumulative wear of subsoiler blade.

1. For the establishment of the geometric model of the optimized subsoiler based on a detailed analysis of the wear conditions of various subsoilers during subsoiling operations, the original bionic micro-spike convex hull subsoiler was used as a foundation. By adjusting the arrangement of the micro-spike convex hulls and introducing a micro-spike scale structure, the new design features an equidistant arrangement on the edge corners of the shovel surface, with six on each side wing and three in the middle, as shown in Figure 11 and Figure 12. This adjustment aims to enhance the wear resistance performance of the subsoiler.

2. Discrete Element Analysis of the Optimized Bionic Subsoiler: The optimized subsoiling device model was exported as a STEP file and subjected to discrete element simulation experiments in the EDEM soil model. Using the discrete element post-processing module, data on the changes in particle speed and force over time, resulting from the traditional shovel tip acting on soil particles, were analyzed and processed, as shown in the figures. The line chart indicates that, after 1 s, the shovel tip advanced in the soil bin at a speed of 1 m/s, and the particle movement speed stabilized. The average total particle speed was 2.399 m/s, with a maximum of 3.797 m/s and a minimum of 1.552 m/s, as shown in Figure 13 and Figure 14. The Relative Wear model was loaded, which records the cumulative tangential energy generated by the interaction between particles and the shovel body. This model can infer the wear location and condition of the shovel tip based on the magnitude and position of the generated energy, as shown in Figure 15 and Figure 16. Within 4 s, the cumulative tangential energy was 0.699 J, with the edge wear of the shovel wings being superior to that of the bionic micro-spike convex hull structure shovel tip.

## 5. Drag Reduction Performance Test of the Optimized Bionic Multi-Subsoiler Implement

### 5.1. Discrete Element Simulation and Experiment

**Figure 17:**
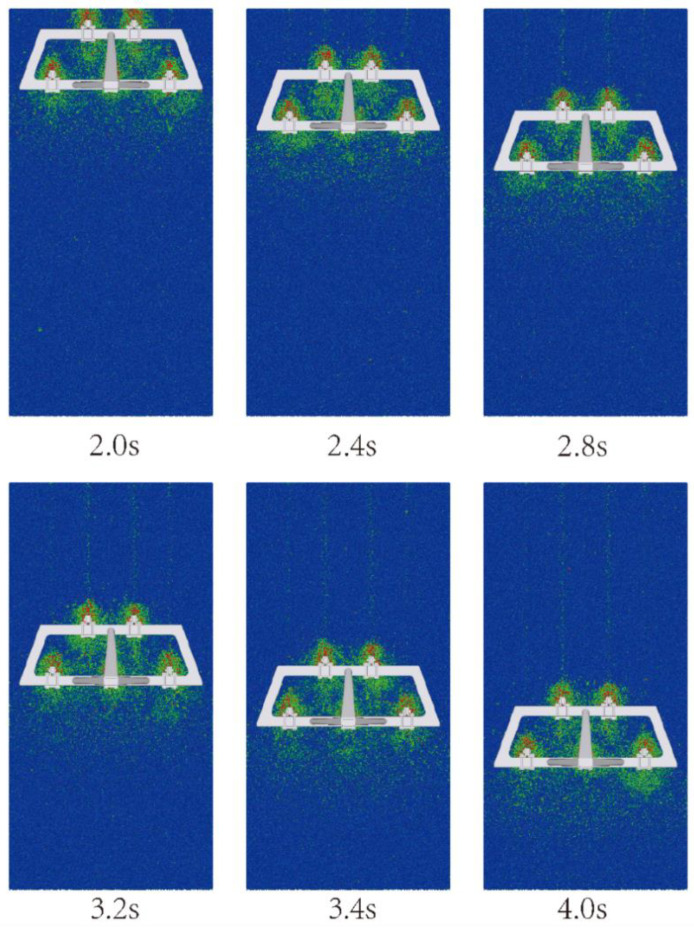
Soil velocity at different time points.

According to the evaluation criteria of the national standard for subsoiling operation quality [28], this experiment uses the soil looseness and soil disturbance coefficients as performance indicators. The study distinguishes soil particles based on whether they have velocity and investigates the soil disturbance effect after the optimized subsoiler operation. By observing soil speed at different positions during subsoiling operations at various times using EDEM, it was found that the soil distribution at different distances after subsoiling is similar, as shown in Figure 17. Therefore, the soil looseness analysis uses the soil section 20 mm behind the plane of the rear subsoiler shank.

The soil cross-section was exported, and the critical curve of the simulated soil particle movement speed was drawn using the profiling method as the pit-shaped contour of soil disturbance. The accumulation contour of the simulated particles on the surface was used as the ridge-shaped contour of soil disturbance. The surface after the initial particle settlement was used as the ground surface contour. The pit-shaped contour of soil disturbance and the ridge-shaped contour of soil disturbance were used as the truncated area from the post-tillage surface to the theoretical subsoiling trench bottom. The pit-shaped contour of soil disturbance and the ground surface contour were used as the truncated area from the pre-tillage surface to the theoretical subsoiling trench bottom. The soil looseness was calculated according to following equation.

(8) $$\begin{array}{l} P=\frac{A_{h}-A_{q}}{A_{q}}\times 100\% \end{array}$$

The response surface experiments reported in this manuscript were conducted using the revised experimental design described herein. A total of 17 randomized runs were performed with specified replications (see Table 2 for run order and replication). Data were analyzed using Design-Expert 13 fitting included the main effects, two-way interactions, and quadratic terms; model diagnostics comprised residual analysis, lack-of-fit testing, and the calculation of R^2^, adjusted R^2^, predicted R^2^, and Adequate Precision. All reported *p*-values are two-sided, and the significance level was set at α = 0.05.

The experimental data is relatively simple and aims to determine the optimal data for the experimental group. Therefore, a Box–Behnken design was established in the Design Expert 12 software. In this study, the maximum subsoiling depth was set at 310 mm based on a comprehensive evaluation of regional agricultural practices, soil mechanical properties, and equipment performances specific to the sloping red soil environment in Southwest China. The rationale for this upper limit includes the following: Soil Characteristics: Red soil in this region exhibits a high adhesion and severe compaction, which exponentially increases operational resistance with depth. Exceeding 310 mm would lead to excessive shear stress (e.g., simulated equivalent stress at 280 mm reached 78.7 MPa in Table 1) and structural fatigue risks for the subsoiler. Crop Requirements: Local crops (e.g., corn, tobacco) typically require subsoiling depths within 250–350 mm to break compacted layers, while avoiding damage to the deeper soil horizons critical for root development. Using a three-factor, three-level approach based on actual conditions, an $L_{1}7(3^{3})$ orthogonal table was selected, with the number of experiments N = 17. Single-factor experiments were conducted with subsoiling speed (0.5, 0.875, 1.25 m/s), entry angle (18.5°, 23°, 27.5°), and entry depth (280, 295, 310 mm). Based on the results of the single-factor experiments, the response surface experiment factors and levels were designed, as shown in Table 2.

Using subsoiling speed, entry angle, and entry depth as response variables, and soil looseness as the response value, experiments were conducted according to the response surface design scheme (Box–Behnken, N = 17). Each experimental run was performed with n = 3 independent replicates; the treatment order was randomized and the mean ± SD is reported for each run. The response values were filled in for data analysis. Based on the data samples in Table 3, the experimental results were subjected to quadratic regression analysis using Design-Expert software. A quadratic function was selected for multivariate fitting, and a quadratic polynomial regression model for soil looseness was obtained by fitting, referencing Zhenwei Tong et al. [28].

Based on the data samples in Table 3, a quadratic polynomial regression model for soil looseness was obtained using Design–Expert software:

(9) $$\begin{array}{l} Y=0.14-0.0157A+0.00437B-7.12C+9.17AB-2.66AC+5.31BC+0.029A^{2}-1.26E-0.4B^{2}-2.06C^{2} \end{array}$$

**Figure 18:**
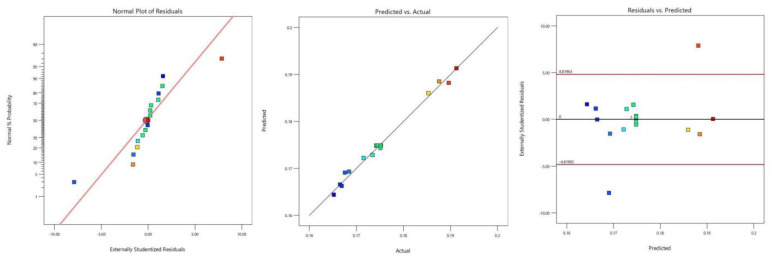
Residual analysis.

Entering the diagnostics section, a series of charts were obtained to evaluate the model’s fit, including the normal probability plot of residuals, the plot of residuals versus predicted values, and the plot of predicted versus actual values, as shown in Figure 18. The normal probability plot of residuals showed a normal distribution, reflecting the model’s good fit to the data. The plot of residuals versus predicted values provided a deeper evaluation of the model’s fit. This plot exhibited no discernible pattern or trend, indicating a good random distribution of residuals versus predicted values. This suggests that the model can uniformly fit the data across the range of predicted values. The plot of predicted versus actual values provided a visual representation of the model’s prediction accuracy. Ideally, this plot should show a distribution close to a straight line, indicating a high consistency between the model’s predicted and actual values. The plot clearly showed a good trend of distribution between the model’s predicted and actual values.

All statistical analyses were performed using Design-Expert 12 and SPSS 27. Prior to hypothesis testing, residual normality was assessed with the Shapiro–Wilk test and homogeneity of variance with Levene’s test. The regression model was evaluated using ANOVA; model terms with *p* < 0.05 were considered significant. Pairwise comparisons were performed using Tukey’s HSD post hoc test (α = 0.05). Model diagnostics included the normal probability plot of residuals, residuals vs. predicted, lack-of-fit test, and R^2^ and adjusted R^2^ reporting. There is only a 0.01% chance that such a large F-value could occur due to noise. A *p*-value less than 0.0500 indicates that the model terms are significant. The soil loosening speed, entry depth, and entry angle were all significant model terms, with soil loosening speed showing the greatest variability. To visually reflect the influence of factors on the determination conditions, interaction effect response surfaces were selected. Based on the response surface methodology, the uncertainty of input variable values and their impact on the results were statistically analyzed using the function relationship. According to the regression model analysis results, the Analysis [+] function of Design-Expert software was used, and 3D Surface views in Model Graphs were selected to plot the interaction effect 3D response surface graphs for the terms AC, BC, and A in the Term column.

The ANOVA for the fitted response surface model (Table 4) indicates a highly significant model (Model F = 285.13, *p* < 0.0001). The model performance metrics are R^2^ = 0.9973, adjusted R^2^ = 0.9938, predicted R^2^ = 0.9389, and Adequate Precision = 53.2731. The model sum-of-squares is 1.100 × 10^−3^ and the residual sum-of-squares is 2.965 × 10^−6^.

Factor significance: Speed (A) is the dominant term (*p* < 0.0001), accounting for approximately 63.64% of the model sum-of-squares. The quadratic term A^2^ is also highly significant (*p* < 0.0001), indicating a strong nonlinearity with respect to speed. The entry angle (B) and working depth (C) are significant (*p* = 0.0021 and *p* = 0.0002, respectively). Interaction A × C is significant (*p* = 0.0464); interactions A × B (*p* = 0.6489) and B × C (*p* = 0.3070) are not significant. The quadratic term B^2^ is significant (*p* < 0.0001), while C^2^ is not (*p* = 0.1028). The lack-of-fit test is not significant (F = 2.97, *p* = 0.1604), indicating the chosen model form adequately captures the systematic variation within the experimental domain.

Model diagnostics were examined through residual analysis. Residuals vs. predicted values and Normal Q-Q plots indicate no major departures from homoscedasticity or normality. Cook’s distance and leverage values were assessed; no influential observations requiring removal were identified. The model therefore meets standard regression assumptions for inference in the tested domain.

**Figure 19:**
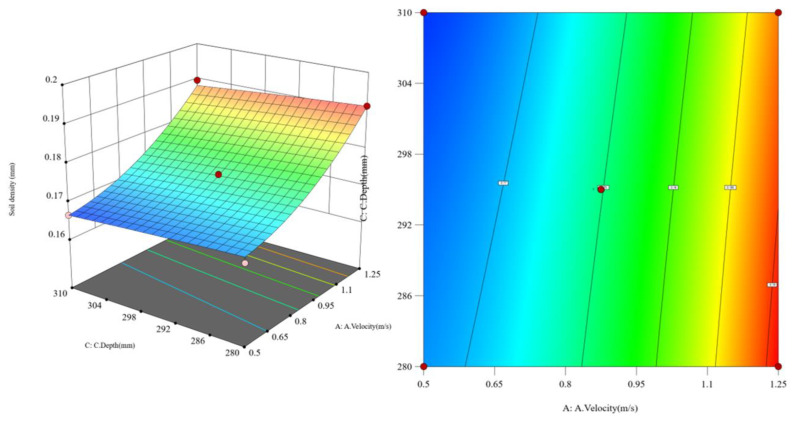
Response surface plot of the interaction between operating speed and subsoiling depth.

**Figure 20:**
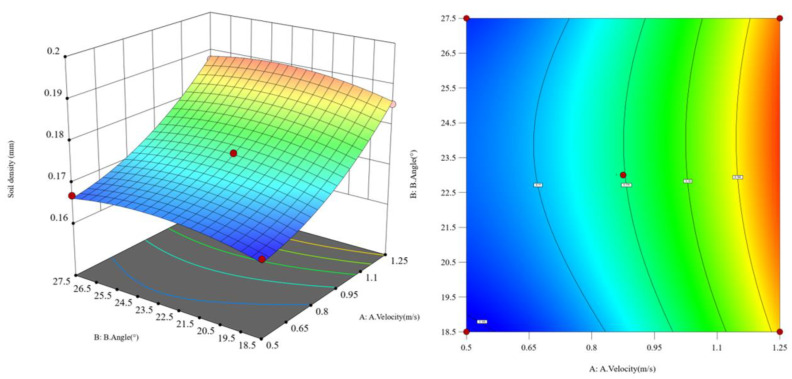
Response surface plot of the interaction between operating speed and subsoiling angle.

**Figure 21:**
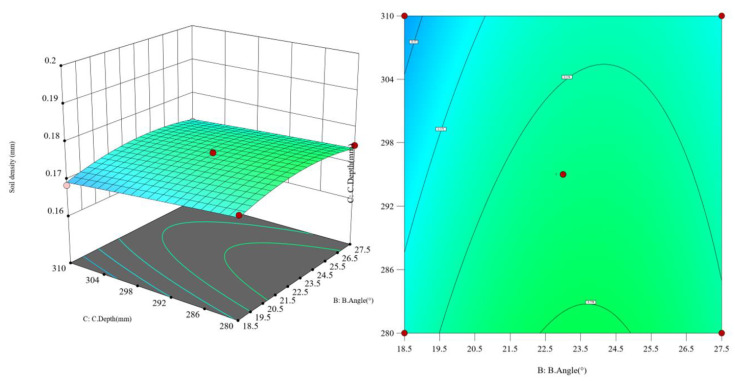
Response surface plot of the interaction between subsoiling angle and operating speed.

Assuming the soil penetration angle is constant, as shown in Figure 19, when the soil penetration depth of the subsoiler decreases from 310 mm to 280 mm, the greater the subsoiling operating speed, and the lower the soil looseness. Assuming the subsoiling depth is constant, as shown in Figure 20, when the soil penetration angle gradually increases from 18.5° to 27.5°, the soil looseness first increases and then decreases. Assuming the operating speed is constant, the greater the subsoiling depth, the lower the soil looseness. However, compared to changing the operating speed, the changes in soil looseness caused by angle variations are more significant. Based on the interaction effect analysis, as shown in Figure 21, when both the subsoiling operating speed and soil penetration depth increase to appropriate values, the effect of reducing the resistance in subsoiling operations is significant, while the soil penetration angle has a smaller effect on reducing the resistance in subsoiling operations.

### 5.2. Parameter Optimization and Comparative Experiments

According to the evaluation criteria of the national standard for subsoiling operation quality [29], after measuring the unplowed surface line, the plowed surface line, and the subsoiling trench bottom line, the cross-sectional area from the pre-tillage surface to the theoretical subsoiling trench bottom and the cross-sectional area from the pre-tillage surface to the actual subsoiling trench bottom were calculated. The soil disturbance coefficient was then calculated using the following equations.

(10) $$\begin{array}{l} y\frac{A_{s}}{A_{q}}\times 100\% \end{array}$$

Statistical analysis: For the soil looseness and disturbance coefficients, we report sample sizes (n), means and sample standard deviations (mean ± SD), and 95% confidence intervals (CI). Standard deviation is computed as formula. The response surface model was validated using ANOVA with reporting of the SS, df, MS, F and P, and partial η^2^ effect sizes. The residual normality and homogeneity of variance were assessed via Shapiro–Wilk and Levene tests; if the assumptions failed, appropriate transformations (log or sqrt) were applied. Simulation vs. soil bin comparisons employed paired t-tests (for near-normal paired data) or Wilcoxon signed-rank tests (nonparametric), with MAE, RMSE, and % relative error calculated. Linear regression (sim vs. exp) and Bland–Altman analyses were performed to quantify the bias and limits of agreement. All tests were two-sided, with α = 0.05. Analyses were performed in Design-Expert 12 and SPSS 27.

(11) $$s=\sqrt{\frac{1}{n-1}\sum (x_{i}-\overset{-}{x}{)}^{2}}$$

The soil bin experiments were conducted at Kunming University of Science and Technology. Red loam collected from cultivated fields in Yunnan Province was used as the test medium. Prior to each trial, the soil bed was compacted to ensure a consistent firmness and to minimize experimental variability arising from soil settlement.

The data acquisition hardware comprised a BELS-2S load sensor paired with a BSFY-1 signal amplifier, an Arduino development board, an SD card module with an SD card, and a laptop. Data acquisition was implemented using the Arduino 2.3.2 software environment: a dedicated acquisition routine was developed to sample the load-cell voltage output during tillage and to convert the measured voltages into traction force values based on the sensor calibration curve. Force readings were monitored via the Arduino serial monitor and logged to the SD card for subsequent processing.

In this study, the biomimetic features were designed so that the surface area occupied by the biomimetic structures was approximately equal to that of the conventional baseline surface, thereby avoiding large biases caused by uneven biomimetic area proportions. To ensure manufacturing accuracy and comparability between specimens, all shovel tips were produced through 3D printing using polylactic acid (PLA). PLA was selected because it offers a good tensile strength, appreciable impact resistance, and is an environmentally friendly material.

**Figure 22:**
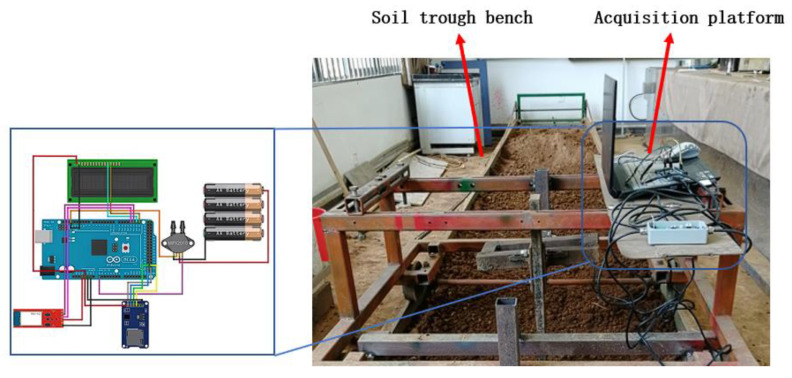
Soil trough experiment.

For the regression models of the soil looseness and soil disturbance coefficients, as shown in Figure 22, the Optimization function in Design Expert software was used for optimization.

**Figure 23:**
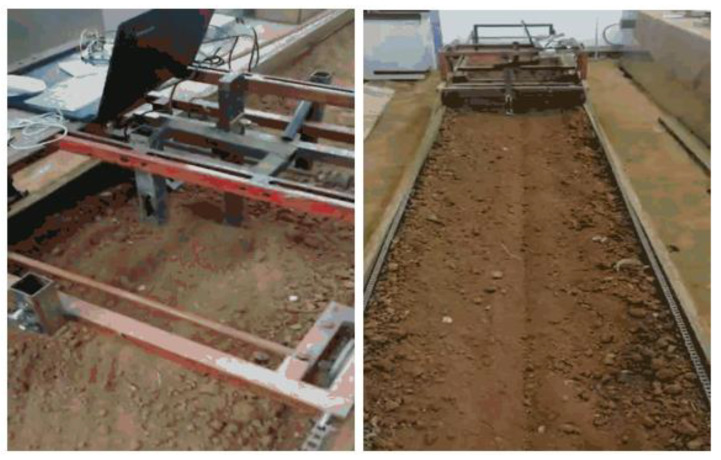
Soil tank experiment process.

By setting the maximization of soil looseness as the target condition and solving the regression model, the optimal parameters for the subsoiler were obtained, including a subsoiling speed of 1.25 m/s, an entry angle of 23.917°, and an entry depth of 280.167 mm. The experimental process is shown in Figure 23.

To verify the reliability of the simulation optimization parameters, EDEM simulations and soil bin comparison tests were conducted under the optimal parameter conditions. Five key points were selected for comparison during the tests. The simulation test results in Table 5 showed that the soil looseness and soil disturbance coefficient were 19.7% and 18.1%, respectively. The soil bin test results indicated that the soil looseness and soil disturbance coefficients were 19.5% and 17.6%, respectively. The comparison revealed that the average error rates between the simulation values and the soil bin test values were 4.385% and 2.76%, respectively, and the trend of soil entry resistance changes in the EDEM simulation test was consistent with that in the soil bin test.

To assess practical relevance, we compared key performance metrics obtained from our soil bin tests and DEM simulations with reported values for subsoilers used in no-till systems. The metrics considered were disturbance intensity (proxied by mean particle speed), soil looseness, and a wear proxy (cumulative tangential contact energy); all metrics were normalized to per-shank or per-unit working width where applicable to ensure comparability. Our composite biomimetic multi-subsoiler shows a clear advantage in disturbance efficiency—the mean particle speed increased from a baseline of 1.52 m/s to 2.399 m/s under comparable test conditions (≈57.8% improvement), while soil looseness results were consistent between the simulation and soil bin validation (simulated 19.7% vs. experimental 19.5%, relative error ≈1.03%), and wear proxies remained within acceptable ranges. We note the inherent limitations of cross-study comparisons (differences in soil type, moisture, rig geometry, and operating conditions); therefore these comparisons should be interpreted as indicative, and we recommend targeted field trials to confirm the actual energy savings and durability benefits under real no-till operating conditions [11,13].

## 6. Conclusions

This study applied engineering bionics to develop four bionic tip geometries/surfaces (convex hull, micro-spike convex hull, scale, micro-spike scale) inspired by *Procambarus clarkii* and *Scincus scincus*. The designs were evaluated using finite element analysis (FEA), discrete element method (EDEM) simulations, and soil bin experiments. The key conclusions are as follows:

1. Mechanical benefits of bionic geometries: FEA demonstrates that the bionic surface geometries reduce the equivalent stress and total deformation compared with the baseline, lowering the risk of structural fracture and improving the bearing capacity—an advantage for operations in viscous red soil.
2. Trade-off between disturbance and wear: EDEM and wear analysis show that the micro-spike convex hull generates the strongest soil disturbance (mean particle speed ≈ 1.92 m/s), while the micro-spike scale generates the lowest cumulative tangential contact energy, indicating a better wear performance. The engineering implication is to adopt a hybrid design: micro-spike convex features for enhancing disturbance and localized micro-spike scales at edges for reducing wear.
3. Optimization and validation: The optimized multi-subsoiler arrangement (micro-spike convex hulls arranged equidistantly along edge corners—six per side wing, three centrally) and operating parameters (speed = 1.25 m/s; entry angle ≈ 23.92°; depth ≈ 280.17 mm) produced consistent results in EDEM and soil bin tests. The reported values are as follows: simulated soil looseness = 19.7%, experimental = 19.5%; simulated soil disturbance = 18.1%, experimental = 17.6%. Using the experimental values as reference, the recalculated relative errors are ≈ 1.03% (looseness) and ≈ 2.84% (disturbance), indicating a good agreement under the tested conditions.
4. Practical significance and scope: The study provides practical geometric and operational guidelines for resistance reduction and wear mitigation in sloping red soil environments. The proposed hybrid bionic surface design achieves a viable compromise between disturbance efficiency and tool longevity.
5. Limitations and future work: The findings are based on laboratory soil bin tests and EDEM simulations; DEM parameter calibration, field variability, and long-term wear/fatigue performance require field validation and extended durability testing. Future work should include field trials across different soil moistures and textures, long-term wear and fatigue studies, and production feasibility and cost–benefit analysis.

## Figures and Tables

**Table 1:** Discrete element method simulation parameters.

Parameter	Numerical Value
Poisson’s Ratio of Soil Particle	0.4
Shear Modulus of Soil Particles/Pa	1.09 × 10^6^
Density of Soil Particles	1350
Poisson’s Ratio of 65 Mn	0.3
Shear Modulus of 65 Mn/Pa	1.92 × 10^6^
Density of 65 Mn/$\left(Kg\cdot m^{3}\right)$	7800
Correlation Coeffcient Between Particles	0.2
Rolling Friction Coefficient Between Particles	0.3
Static Friction Coefficient Between Particles	0.4
Correlation Coefficient Between Particles and Subsoiler	0.3
Rolling Friction Coefficient Between Particles and Subsoiler	0.4
Static Friction Coefficient Between Particles and Subsoiler	0.5
Particle Radius/mm	4
Particle Mass/Kg	2400
Gravitational Acceleration/$\left(Kg\cdot m^{3}\right)$	9.81
Simulation Time Step/s	4

**Table 2:** Finite element analysis results of various subsoiler groups.

Code	Name	Organization	Min	Max
A	Speed	m/s	0.5	1.25
B	Angle	°	18.5	27.5
C	Depth	mm	280	310

**Table 3:** Levels of various factors.

Std	Run	$A/(m\cdot s_{1})$	B/(∘)	C/(mm)	Soil Looseness/(%)
17	1	0.875	18.5	280	0.173456
9	2	1.25	23	310	0.189655
5	3	0.875	23	295	0.174231
1	4	0.5	23	295	0.167574
14	5	0.875	23	295	0.174514
3	6	0.875	27.5	310	0.171546
10	7	0.875	23	295	0.174555
7	8	1.25	18.5	295	0.185316
2	9	0.5	18.5	295	0.165214
13	10	0.875	23	295	0.175315
4	11	0.875	23	295	0.175239
12	12	0.875	18.5	310	0.168445
15	13	0.5	23	310	0.166516
8	14	0.875	27.5	280	0.175123
11	15	1.25	23	280	0.191302
16	16	0.5	27.5	295	0.166894
6	17	1.25	27.5	295	0.187615

**Table 4:** Data source.

Source of Variance	Sum-of-Squares	Degrees of Freedom	Mean Square	F	*p*
Model	0.0011	9	0.0001	285.13	<0.0001
A	0.0007	1	0.0007	1747.37	<0.0001
B	9.56 × 10^−6^	1	9.56 × 10^−6^	22.58	0.0021
C	0	1	0	54.13	0.0002
AB	9.58 × 10^−8^	1	9.58 × 10^−8^	0.2262	0.6489
AC	2.47 × 10^−6^	1	2.47 × 10^−6^	5.83	0.0464
BC	5.14 × 10^−7^	1	5.14 × 10^−7^	1.21	0.307
A2	0.0001	1	0.0001	186.64	<0.0001
B2	0	1	0	82.79	<0.0001
C2	1.49 × 10^−6^	1	1.49 × 10^−6^	3.52	0.1028
Residual	2.97 × 10^−6^	7	4.24 × 10^−7^		
Lack-of-fit Term	2.05 × 10^−6^	3	6.82 × 10^−7^	2.97	0.1604
Pure Error	9.19 × 10^−7^	4	2.30 × 10^−7^		
Sum	0.0011	16			

**Table 5:** Comparison of simulation and experimental results under optimal parameters.

Serial Number	Soil Looseness %		Simulation	Soil Disturbance Coefficient %		Simulation
	Experimental	Simulation		Experimental	Simulation	
1	0	0	0	5 × 12.7	5 × 18.1	5 × 2.76
2	5.3	5.5	3.63			
3	9.7	10.1	3.96			
4	13.1	14.3	8.93			
5	19.5	19.7	1.01			

## Data Availability

The original contributions presented in this study are included in the article. Further inquiries can be directed to the corresponding author.
